# Excellent antibacterial and anti-inflammatory efficacy of amoxicillin by AgNPs and their conjugates synthesized using *Micromeria biflora* crude flavonoid extracts

**DOI:** 10.1016/j.heliyon.2024.e36752

**Published:** 2024-08-23

**Authors:** Kamran Jalil, Shabir Ahmad, Nazar ul Islam, Sayyar Muhammad, Qudsia Jalil, Asad Ali

**Affiliations:** a Department of Chemistry, Islamia College, Peshawar, 25120, Khyber, Pakhtunkhwa, Pakistan; b Department of Pharmacy, Sarhad University of Science & Information Technology, Peshawar, Khyber, Pakhtunkhwa, Pakistan; c Energy Engineering, Division of Energy Science, Lulea University of Technology, 97187, Lulea, Sweden

**Keywords:** Ag-amoxi conjugates, Amoxicillin, Anti-inflammatory, Antimicrobial, Antinociceptive, *Micromeria biflora*

## Abstract

Antibacterial resistance is considered to be one of the major causes for mortality in coming years. In recent years green nanotechnology played a key role in addressing this problem. Biocompatible metal nanoparticles have gained popularity owing to their excellent therapeutic effects and minimal side effects.

**Method:**

We report the synthesis of AgNPs and their amoxicillin conjugates (Ag-amoxi) using *Micromeria biflora* crude flavonoid extracts. The physicochemical properties of the synthesized NPs and Ag-amoxi conjugates were systematically evaluated using scanning electron microscopy (SEM), energy dispersive X-ray (EDX) and X-ray diffraction (XRD) analysis, Fourier transform infrared (FTIR), and UV–visible (UV–Vis) spectroscopic techniques.

**Results:**

The average sizes of AgNPs and Ag-amoxi conjugates were 45 and 62 nm, respectively. We have also explored the antibacterial, antioxidant, anti-inflammatory, and analgesic properties of the AgNPs and Ag-amoxi conjugates through in *vivo* and in *vitro* analysis. The Ag-amoxi conjugates showed better antibacterial potential against *Streptococcus Pneumoniae* (S.P), *Staphylococcus aureus* (S.A), *Pseudomonas aeruginosa* (P.A), and *Methicillin resistance Staphylococcus aureus* (MRSA) strain both the drug and AgNPs. Similarly, in *vivo* anti-inflammatory studies revealed that both Ag-amoxi (68 %) and AgNPs (64 %) had strong anti-inflammatory effects, with (***p < 0.001) significance at a dose of 10 mg kg^−1^ body weight as compared to standard, amoxicillin (45 %), and flavonoids extract (48 %) at a dose of 100 mg kg^−1^. The findings of the antinociceptive activities (writhing and hot plate tests) demonstrated that the Ag-amoxi conjugates produced fewer writhing (15 in 20 s) and a shorter latency time of 22 s as compared to vehicle-treated (tramadol) animals, amoxicillin, and P.E at much lower doses. In *vitro* antioxidant studies revealed that the Ag-amoxi conjugate has the potential to be used as an antioxidant with an IC_50_ value of 43.58, compared with AgNPs (46.34), amoxicillin (58.17), compared to the standard of ascorbic acid (34.14).

**Conclusion:**

These results reveals that these biologically inspired AgNPs and Ag-amoxi conjugate could be used to improve antibiotic efficiency and could play a critical role in addressing the multidrug resistance problem in coming years.

## Graphical abstract

**Figure:**
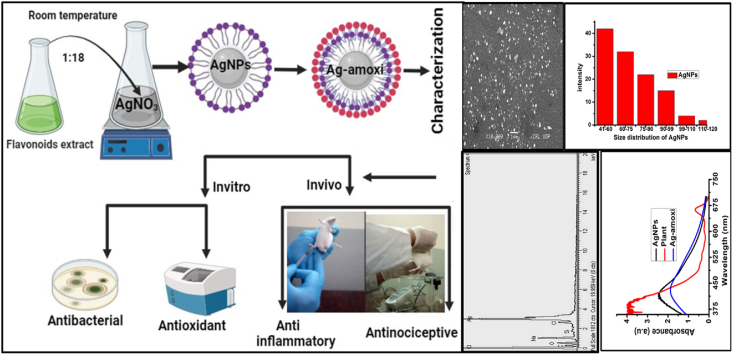

## 1 Introduction

Modern medicines have been advanced by nanotechnology, the biggest engineering innovation in recent times [1].The demand for items made by nanotechnology is growing daily. The cutting-edge technology of today, can significantly influence how people's health can be improved [2].Nanoparticles unique physicochemical properties, as well as their improved performance, strength, durability, and flexibility, have been studied in the health sector [3]. Green nanotechnology emphasizes on the synthesis of eco-friendly nanomaterial using bioactive substance extracted from plants or other natural material. Several studies have been reported where the plant material has been used for the synthesis of AgNPs which presented with good antibacterial, antifungal and antioxidant properties [4]. Medicinal plants contain a high percentage of chemical constituents that cause changes in the physiological conditions of the human body to prevent diseases [5]. In the course of nanoparticle synthesis, either whole plants or specific parts such as fruits, leaves, stems, and roots, have been used. The rich phytochemicals present in the plant extracts are used as reducing, capping, and stabilizing agents. Several reports have been published on the synthesis of AgNPs using green pathways such as *Lysiloma acapulcensis* [6], *Berberis vulgaris* [7]*, Bunium Persicum* [8]*, Duchesnea indica* [9]*, Acacian Ilotica* [10]*, Cestrumnocturnum* [11]*, banana peels* [12], *Dodonaea viscosa* [13], *Lallemantia royleana* leaf Extract [14], *Otostegia persica (Burm.) Boiss.* leaf extract [15], and *P. domestica* extract-mediated AgNPs can be used for nociceptive pain management in children [16]. In the last few years, nanoscale technology has been related to drug conjugation to enhance their efficacy against many common and resistant pathogens. It has been reported that when drug molecules are encapsulated in lipid-coated polymeric nanoparticles the cytotoxicity of the drug can be improved [17]. Green AgNPs have shown low toxicity against normal cells [18]. Both gold and silver have been used as the basis for biocompatible NPs with varying results” Nikparast studied the synergetic effect of biocompatible AgNPs and used them in conjunction with ciprofloxacin, a broad-spectrum antibiotic that shows excellent antimicrobial effects along with AgNPs [19]. Mocan,L et al. reported that AuNPs conjugated with vancomycin can bind to the protein portion of the pathogen cell wall and inhibit the growth of pathogenic bacteria [20]. Muenraya et *al.* [21] reported that AgNPs conjugated with colistin, a protein peptide antibiotic, showed excellent activity against gram-positive bacteria. Abdul Kader Masri et al. [22] reported that nano antibiotic conjugates and small molecule capped NPs can be used to enhance the efficacy of antibiotics. Tayagi et al. [23] published their research showing that chemically induced ZnO nanoparticles when conjugated with ciprofloxacin showed excellent antimicrobial activities against common pathogens. *Tinospora cordifolia* mediated AuNPs have been used against resistant strain of *p. aeruginosa* [24]. AgNPs prepared by chemical means, conjugated with amikacin shows excellent antibacterial activities [25]. Additionally, the activity of doxorubicin towards cancerous cells was found to be increased when conjugated to Zn-NPs that were synthesized using the plant pathogenic fungus *Aspergillus niger* [26]

*H.Vahidi* et al. synthesized selenium nanoparticles using penicillium chrysogenum and reported excellent antibacterial activities [27]. Similarly studies were carried out by Golnaraghi Ghomi AR et al. using pencillinium fungus species for synthesis of Zirconium NPs shows excellent antibacterial properties [28]. *S*. *Majeed* et al. used Green-synthesized TAT peptide-functionalized silver nanoparticles for apoptotic cell-death mediated therapy of breast carcer [29].

Micromeria biflora belongs to family Lamiacea and have been used in the herbal remedies since ancient times. It has been reported that Micromeria can be used for headache, fever, cold, skin infections, and wounds healing [30]. More than 100 species of Micromeria are available all over the world. However, only three species are available in the Himalayan region [31]. Micromeria species are also reported to have many pharmacological activities such as anti-rheumatic, antiseptic, and anesthetic and the extracts of some species are reported that have shown antioxidant, anticholinesterase antibacterial, antifungal, insecticidal activities [32]. It has been reported that 100g of Micromeria biflora contains 20 % of flavonoids, 3.02 % of Saponin and 8.90 % of Alkaloids [33].

Amoxicillin is a well-known and commonly prescribed antibiotic. It is antibacterial and effectively combats most gram-positive bacteria. However, in recent years, some common bacterial strains have developed resistance towards it. One such example is *Helicobacter pylori*, which causes gastric cancer in advanced countries [34]. Previously, amoxicillin-coated AuNPs have been used to study photo-induced antibacterial activities against resistant *Staphylococcus aureus* strains [35]. A similar finding was reported by Kalita et al. [36] who used microwave-assisted biogenic AuNPs functionalized with amoxicillin to reverse MRSA antibacterial resistance. Similarly, chemically synthesized AgNPs using amoxicillin as a stabilizing agent can be used to develop not only different therapeutic tools but can also increase the antibacterial efficacy of the amoxicillin [37]. Plant mediated AgNPs got recognition in last few years due to their non toxicity and eco-friendly nature. During NPs synthesis bioactive compounds such as flavonoids, phenols, citric acid, ascorbic acid, polyphenolic, terpenes, alkaloids and reductase that use as reducing and stabilizing agent [38,39].

In the present study we report the synthesis of AgNPs using crude *flavonoids* extract from *Micromeria biflora* adopting a simple cost-effective approach. The synthesized AgNPs were subsequently conjugated with amoxicillin in a single step without the use of any additional linkers/polymers. As for as we know all the reported literature involving conjugation of drugs requires extensive use of additional polymers or linkers for their stability. Therefore, our work is significance in this regard since amoxicillin was conjugated with synthesized AgNPs in a single step without using any further linkers or polymers. The prepared AgNPs and silver-amoxicillin (Ag-amoxi) conjugates were characterized using SEM, XRD, EDX, UV–visible and FT-IR spectroscopy. Moreover, the compounds were screened for their antibacterial, anti-inflammatory, anti-oxidant and antinociceptive activities. The use of plants extract for the synthesis of NPs has got wide recognition for the last few decades. Bioactive compounds such flavonoids, phenols, citric acid, ascorbic acid, polyphenolic, terpenes, alkaloids and reductase present in plants are responsible for reduction and capping of NPs [39]. Thus, in the present work the crude flavonoids extract of *Micromeria biflora* was used for the formation of AgNPs, which were subsequently conjugated with amoxicillin for their excellent antibacterial, anti-inflammatory and antinociceptive properties. The phytochemicals present in plant extracts have the ability to act as reducing agents and give stability to the AgNPs and affect their physicochemical properties a great deal [40].

## 2 Material and methods

*2.1: Plant collection***:***Micromeria biflora* (*M. Biflora*) plant was collected from a small valley Bishbanr on Malam Jabba Road District Swat Malakand division of Khyber Pakhtunkhwa in the month of June. Morphology and specie were confirmed by Professor Amaan Ullah an Associate Professor of Botany at Govt Degree College Hayatabad Peshawar (Pakistan). A specimen of the plant was kept in herbarium of the Botany Department of Govt. Degree College Hayatabad Peshawar with voucher No GDCH M.B-2020.

### 2.1 Chemicals

Silver nitrate, AgNO_3_ (99.8 % pure), Barium chloride Dihydrate, BaCl_2_.2H_2_O (99.9 % pure), sulphuric acid, H_2_SO_4_ (98 % pure), acetic acid, CH_3_CO_2_H (96 % pure) were purchased from Merck (Germany). Amoxicillin was purchased from GlaxoSmithKline (GSK) Pharmaceuticals. The 2, 2-diphenyl-1-picrylhydrazyl (DPPH) kit was purchased from Sigma–Aldrich. Agar medium (1–2%) were purchased from LabMal Malaysia. Double-deionized water was used throughout the experiments. All the chemicals were used as received without any further purification processes.

Antibacterial activities were assessed in the Department of Microbiology at the Institute of Biomedical Science, Khyber Medical University Peshawar. Gram-positive bacterial strains of *S. aureus (S.A)*, *Streptococcus pneumoniae, methicillin-resistant S. aureus (MRSA),* and gram-negative *Pseudomonas aeruginosa* (P.A) were used.

In vivo anti-inflammatory and analgesi*c* activities were assessed at the Department of Pharmacy, University of Peshawar using healthy BALB/c mice of either sex (weight. 25–30 g) maintained in metal cages for seven days (22 ± 2 °C with a 12 h light/dark cycle). They had unlimited access to food and drinks. The study protocol was approved by the institutional ethics committee (application number 09/EC/F) LIFE-2020.

### 2.2 synthesis of AgNPs using crude flavonoids extract of *Micromeria biflora*

AgNPs were prepared using the classical Turkevich method [41] with slight modifications, using crude *flavonoid* extract of *Micromeria biflora* 1 mM AgNO_3_ solution was prepared and stirred at room temperature for 1 min, followed by subsequent addition of crude flavonoids extracted from *Micromeria biflora* and the mixture was stirred again for 4 h. The solution's colour changed from pale yellow to dark brown soon after adding the extract, indicating the formation of AgNPs. Different volume ratios (5:1, 10:1, 15:1, 18:1, and 20:1) of 1 mM AgNO_3_ solution and crude *flavonoids* extracts were used for obtaining various shapes and sizes of NPs. Based on maximum absorbance obtained by UV–visible analysis, (18:1) was selected as optimal ratio of AgNO_3_ and plant extract which was confirmed by dark brown colour of the solution and UV–visible spectra.

### 2.3 synthesis of Ag-amoxi conjugates

A modified procedure was adopted [42] to obtain Ag-amoxi conjugates. 10 mL of 1 mM AgNPs were placed in a 100 mL Erlenmeyer flask, 3 mL of 1 mM *amoxicillin* solution was added, and the mixture was stirred for 72 h at room temperature. The mixture was then centrifuged at 18,000 rpm 15–20 min and the resulting pellet was suspended in deionized water to remove unbound drug molecules. The formation of Ag-amoxi conjugates was confirmed from UV–vis spectra.

### 2.4 Stability of AgNPs

The stability of the AgNPs was checked by applying parameters, such as concentration (volumes), pH, and temperature.

### 2.5 Characterization of AgNPs

The characterization of AgNPs was performed using standard characterization techniques as described. The surface morphologies were confirmed with SEM (JSM 5910 JEOL, Tokyo, Japan). The elemental composition of AgNPs and Ag-amoxi was confirmed by EDX (INC-200, Oxford Instruments, Abingdon, UK), while the crystalline structure was confirmed with XRD (JDX-9C-XRD, Tokyo, Japan) The XRD analysis was done at 2θ ranging from 10° to 70° with Cu_kα_ wavelength 0.15406 nm radiations at room temperature. The step interval was kept at 0.045°, with a scan rate of 4.5° per min. The tube current was 30 mA, and the generator voltage was kept up to 40 kV. The various functional groups identification in the plant extract, *amoxicillin*, AgNPs and Ag-amoxi was carried out using FT-IR (Prestige 21 Shimadzu, Kyoto, Japan). UV–visible spectroscopy serves as the most common non-destructive method used for characterization of NPs and their conjugates. The synthesis of AgNPs and Ag-amoxi conjugates were also confirmed by analyzing surface plasmon resonance (SPR) peaks using UV–Vis spectrophotometry (Hitachi U-3200, Tokyo, Japan).

### 2.6 Antibacterial assays

The antibacterial efficacy of the compounds was examined using the agar-well diffusion method. Clinical isolates of *Staphylococcus aureus*, *Streptococcus pneumoniae*, *Pseudomonas aeruginosa*, and *methicillin-resistant Staphylococcus aureus* were used in this study. The initial medium was created by combining 0.4 g of nutritional broth with 50 mL deionized water, pH was adjusted at 7.0 and autoclaved. Mueller-Hinton Agar medium (MHA) was prepared by combining 2.3 g of nutritional agar medium with 100 mL of distilled water. pH of was adjusted at 7.0 and was autoclaved at 121 °C. Following autoclaving, the medium was transferred to a Petri dish. 0.5 McFarland Standard was created by mixing 0.05 mL of 1.175 % BaCl_2_ dihydrate (BaCl_2_.2H_2_O) with 9.95 mL of 1 % sulphuric acid. One day before the experiment, the bacteria were grown in a suspension of nutrient broth medium. After adjusting to a McFarland standard of 0.5, the bacterial inoculum was distributed onto MHA plates. Then 4 mm wells were created in the medium. The Petri plate wells received (100 μL) of the test chemicals, which were then added and incubated for 24 h at 37 °C. The tests were performed twice. After 24 h, zones of inhibition of the test drugs were assessed. The results were noted and interpreted accordingly.

### 2.7 Antioxidant assay

The *DPPH* (2,2-diphenyl-1-picrylhydrazyl) radical scavenging assay was used to evaluate the capacity of the extract to neutralize free radicals [32]. The ability of plant extracts to liberate hydrogen atoms was assessed by measuring the color of a solution of DPPH in methanol. The color of DPPH in methanol solution changes from purple to yellow as it is quenched by antioxidants. For this experiment, methanol extracts containing different doses (31.5–1000 g mL^−1^) of AgNPs, Ag-amoxi, plant extract, and *amoxicillin* solutions, and 2.4 mL of a 0.1 mM DPPH solution in methanol were combined. After vigorous vertexing the reaction mixture was incubated at room temperature for 30 min. The percentage of scavenger activity was calculated using Equation 1

(1) $$\%\;\text{DPPH}\;\text{radical}\;\text{scavenger}\;\text{activity}=\frac{\left(A_{o}-A_{1}\right)}{A_{o}}\times 100$$

where A_o_ is the control absorbance and A_1_ is the extract absorbance/standard AgNPs, Ag-amoxi, and *amoxicillin*. The percentage inhibition was plotted against the concentration, and the IC_50_ values were calculated from the graph. The experiment was repeated 3 times for each concentration.

### 2.8 Anti-inflammatory assays

Anti-inflammatory activity was determined according to the standard protocol by Carrageenan hind paw method [43]. The mice were split up into six sets, each of which had six animals. *Set*I animals received carrageenan as negative control along with a vehicle dose of 3 % DMSO, 1 % Tween-80, and 96 % normal saline. Diclofenac (50 mg kg^−1^) was given to SET II as a positive control, and the other Sets (III–VI) received dosages of plant extract (50 mg kg^−1^ and 100 mg kg^−1^), *amoxicillin* (50 mg kg^−1^ and 100 mg kg^−1^), and AgNPs and Ag-amoxi conjugates (5 mg kg^−1^ and 10 mg kg^−1^) respectively. After 60 min of therapy, they received an injection of carrageenan (0.05 mL; 1 %) solution under the left hind paw. The paw volume was measured using a digital plethysmometer (Plan lab, Spain) at various time intervals (1h, 3h, and 5 h). Percentage inhibition was calculated using Equation (2).

(2) $$\%\;Inhibition=\frac{A-B}{A}\times 100$$

where A, and B are the increase in paw volume of control and test treatment sets, respectively.

### 2.9 Antinociceptive activities

This activity was performed in albino mice of both sexes through acetic acid induced writhing test and the hot plate test.

#### 2.9.1 Acetic acid induced writhing test

In this investigation, albino mice of both sexes weighing 18–22 g were used. Prior to the test, all animals were fasted for 2 h. The total number of animals were divided into groups of six (n = 6). Group I received an intraperitoneal injection (i.p) of a vehicle as a control; group II received a normal medication, diclofenac sodium (20 mg kg^−1^ body weight); and the other groups received intraperitoneal injections of AgNPs and Ag-amoxi (5 and 10 mg kg^−1^, i.p.), *amoxicillin* (50 and 100 mg kg^−1^, i.p.), and flavonoids extract. (50 and 100 mg kg^−1^, i.p.). After 30 min. 1 % acetic acid intravenous injection was administered to the animals. The writhing was measured 5 min after the acetic acid injection. The number of abdominal constrictions (writhes) were recorded for 20 min. The number of writhes used to compute the percentage antinociceptive effect was determined using Equation (3).

(3) $$\%\;Inhibition=\frac{1-test}{Vehicle}\times 100$$

#### 2.9.2 Hot plate test

A hot plate test was used to observe the nociceptive pain in rats and evaluate the efficacy of analgesic drugs by measuring the latency of the animal’s reaction to the thermal stimulus. A pain induced effect of *amoxicillin*, AgNPs, Ag-amoxi and flavonoid extract was determined in mice using a hot plate analgesiometer set at 54.0 ± 0.10 °C. The animals were split into six groups: Group I was treated with vehicle (10 mL kg^−1^) dose, Group II was treated with a common analgesic Tramadol at a dose of (30 mg kg^−1^), while Group III-VI were treated with test compounds AgNPs, Ag-amoxi, (5 and 10 mg kg^−1^) *amoxicillin* and flavonoids extract (50 and 100 mg kg^−1^) respectively. The tramadol, and test substances were injected intraperitoneally to the vehicle. The 30-s post-treatment cutoff was used. At intervals of 30, 60, and 90 min the animal's withdrawal response on the hot plate were observed. Based on the latencies, the percentage of antinociceptive activity was determined by using Equation (4).

(4) $$\%\;protection=\frac{test-baseline}{cutoff-baseline}\times 100$$

##### 2.9.2.1 Statistical analysis

Data were analyzed by one-way ANOVA followed by Tukey’s test for inflammatory and antinociceptive activities while Duncan’s test for antibacterial activities using Graph pad prism software. A *p-*Value of ≤0.001 and p < 0.05 were considered statistically significant.

## 3 Results and discussion

AgNPs were successfully synthesized by the aforementioned method and obtained as a typically yellow-brown colored solution containing the AgNPs [44]. The collective excitation of free electrons on the surface of nanoparticles, known as surface plasmon resonance, was responsible for the colour observed in the solution [45]. When a solution containing silver ions is treated with reducing agent, such as the *flavonoid*s extract in this case, the silver ions are reduced to metallic silver, which aggregate to form nanoparticles [46]. In the second step, these AgNPs were treated with (1 mM) *amoxicillin* to obtain Ag-amoxi conjugates according to reported literature [42] with a slight modification.

Different spectroscopic and analytical techniques such as SEM, EDX and XRD analysis, FTIR and UV–Vis spectroscopy as described in the experimental section were used to confirm the synthesis of AgNPs and their Ag-amoxi conjugates and get information about their morphology, shape, size, stability, functional group and wavelength of maximum absorbance.

### 3.1 Characterization of AgNPs and Ag-amoxi conjugates

**Fig. 1:**
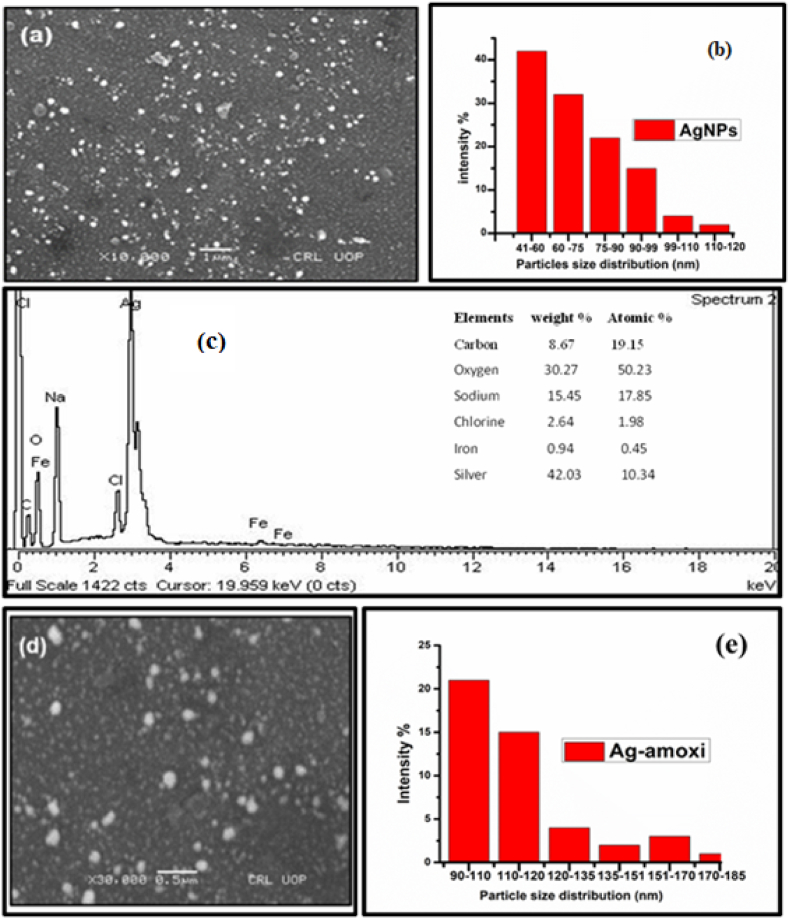
Displays (a) SEM image, (b) particle size distribution and (c) EDX of AgNPs, and (d) SEM image and (e) particle size distribution of Ag-amoxi conjugate.

SEM was used to investigate the morphology, shape and size of the AgNPs and Ag-amoxi conjugates. The SEM obtained for the AgNPs covered by flavonoids extract, Fig. 1 (a), shows spherically shaped AgNPs with different size distributions. Similarly, the SEM image obtained for drug conjugates (Ag-amoxi) also reveals the spherical nature of the Ag-amoxi conjugates albeit with greater diameters (Fig. 1 (d)) indicating much larger sizes compare to the AgNPs**.**

The dimeter histogram obtained for AgNPs (Fig. 1 (b)) shows that the size of the AgNPs ranged from 39 nm to 120 nm with an average size of 45 nm. The salt AgNO3 is reduced by the flavonoid’s hydroxyl moiety from the +1 to zero oxidation state [47]. Similarly, the size distribution histogram of Ag-amoxi conjugates (Fig. 1 (e)) shows that their size distribution ranges from 40 nm to 167 nm with an average size of 62 nm. The increase in the average size of the Ag-amoxi conjugates is attributed to the aggregation of NPs during exchange of some of the flavonoid’s molecules with *amoxicillin* molecules [48].

EDX analysis is widely used to determine the elemental composition of samples, including AgNPs synthesized through various methods [49]. The presence of Ag and other elements was confirmed along with their percentage compositions by EDX as shown in Fig. 1 (c). The result showed that Ag is the dominant element in the analysis with a prominent peak at 2.9 keV in the EDX spectrum, having 42.03 % Ag by mass, with the elements Na, Cl, C, O and Fe also giving strong signals as evident from the table inserted in Fig. 1 (c). These elements may be due to the biomolecules present in the extract [50].

**Fig. 2:**
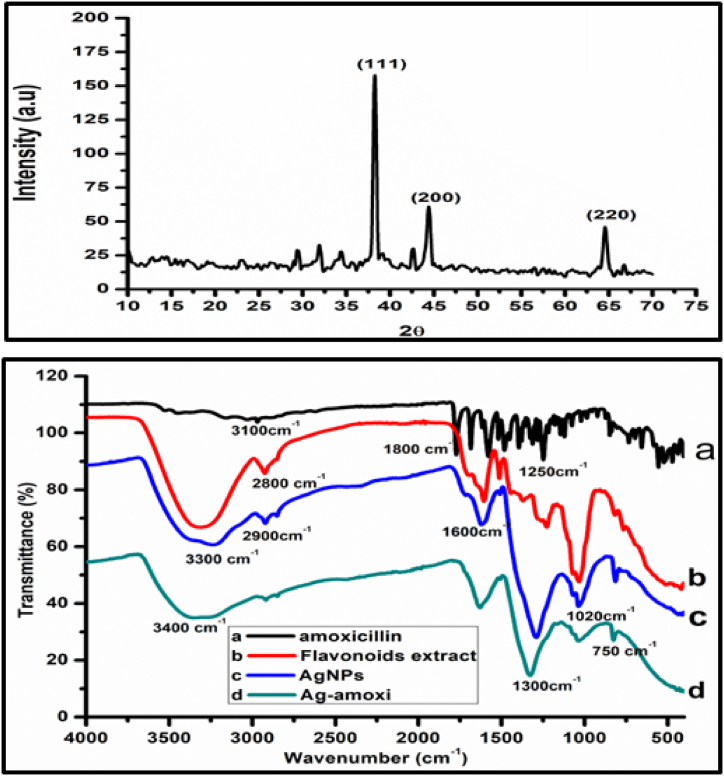
Shows (a) XRD analysis of AgNPs and (b) comparative FTIR analysis of AgNPs, Ag-amoxi, penicillin and plant extract.

Fig. 2 (a) shows the XRD spectrum of the synthesized AgNPs. Prominent peaks can be observed at 2θ = 38.75°, 44.75°, 64.00° designated with hkl Miller indices at (111), (200) and (202), respectively. These hkl indices are due to AgNPs (Match-Phase 96-150-9147). The XRD study showed that the AgNPs had crystalline structure with cubic shape and in good agreement with reported literature for AgNPs [51].The most prominent diffraction peak in the spectrum was used in Scherer’s equation to calculate the crystallite size as given in Equation (5).

(5) $$D=\frac{K\lambda }{\beta \;\cos\;\theta }$$

where D is crystal size in nm, K is Scherer’s constant (0.9), λ is the wavelength of X-ray source (0.15406 nm), β is the half-width of the peak in radians and θ is the corresponding peak position in radians [52]. The crystallite size was calculated to be 42.04 nm.

Formation of AgNPs using flavonoids extract from *Micromeria biflora* have also been confirmed by FTIR spectroscopy. Fig. 2 (b) displays the FTIR spectra of AgNPs (blue line) and a comparison of FTIR spectra of *amoxicillin* (black line), Ag-amoxi (green line), and flavonoids extract (red line). The FTIR spectra of the *amoxicillin* spectrum shows a wide band at 3100 cm^−1^ which is attributed to the presence of hydrogen-bonded OH or NH_2_ group. When the *amoxicillin* is conjugated to the AgNPs this band is sharper and appears at 3300 cm^−1^ in Ag-amoxi. The extract exhibits significance absorption bands at 3200 cm^−1^ (O-H stretching), 2900–2850 cm^−1^ (C-H stretching), 1608 cm^−1^ (C=O stretching), 1500 cm^−1^ (C=C stretching), 1350 cm^−1^ (C-H bending), 1210 cm^−1^ (C-O stretching), and small bands in the region of 500–1000 cm^−1^(aromatic C-H out of plane bending), which are the characteristic absorption bands for phenolic compounds (flavonoids) [53]. The FTIR spectra of plant mediated AgNPs showed bands at 3450 cm^−1^ (O-H stretching), 2950 cm^−1^ (C-H stretching), 1650 cm^−1^ (O-H stretching), 1500 cm^−1^ (C=C aromatic stretching), 1350 cm^−1^ (C-H bending), 1110 cm^−1^ (C-O stretching), and small bands in the region of 500–1000 cm^−1^ (aromatic C-H out of plane bending). While, AgNPs conjugates with amoxicillin have observable changes in the intensities of the absorption peaks (OH sterching frequencies) can be seen in Fig. 2b. A number of changes can be observed from 400 cm^−1^–2000 cm^−1^ in *amoxicillin* spectra when the amoxicillin was conjugated with AgNPs [54]. Ag-amoxi showed a much lower intensity peak at 3500 cm^−1^ which might be due to hydrogen bonding of NH_2_ of *amoxicillin* with O–H of flavonoid responsible for capping of Ag-amoxi.

### 3.2 UV–visible spectra and stability of AgNPs

**Fig. 3:**
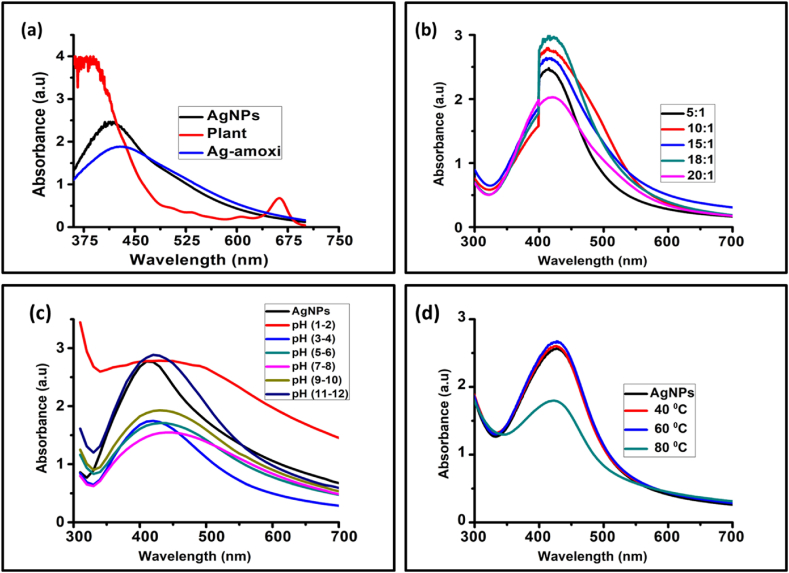
UV–Vis spectra of (a) AgNPs and Ag-amoxi (b) showing effect of concentration, (c) pH and (d) temperature on their stability.

Different volumes of AgNO_3_ (1 mM) were used against fixed volume (1 mL) of crude flavonoids extract of *Micromeria biflora* to reduce Ag ^+^ ions to Ag^0^. The UV–visible spectra for the plant extract, AgNPs and Ag-amoxi are shown in Fig. 3. It is evident from Fig. 3 (a) that a maximum absorption peak at *λ*_max_. = 423 nm at 18:1 v/v concentration ration for AgNPs is observed. A similar *λ*_max_ of AgNPs has been previously reported by *Shah* et al. [10]. The formation of AgNPs was constantly monitored through a colour change in the reaction mixture from colorless to dark brown. The change in colour is attributed to collective excitation of free electrons on metal surface [55]. In addition, the UV–visible spectra of crude flavonoid extract of *Micromeria biflora* confirms that the extract does not contribute to the peak in the same region as it shows only a peak at *λ*_max_. = 664 nm. Similarly, the formation of Ag-amoxi conjugates was also confirmed with UV–visible spectroscopy. The absorption peak at *λ*_max_. = 430 nm indicates formation of Ag-amoxi conjugates. The shifting of the absorption band of Ag-amoxi conjugates towards longer wavelength compared to AgNPs may be attributed to replacement of plant moieties (flavonoids) by *amoxicillin* [42].

The AgNPs generation is dependent on the concentration of the AgNO_3_ solution. Decrease productivity of AgNPs formation is suffered the outcome of nanoparticle formation at higher concentrations [56]. However, studies found that specific conditions and compositions, such as a ratio of AgNO_3_ and plant extract, can lead to optimal synthesis outcomes without necessarily requiring high AgNO_3_ concentrations [57]. Our results are also consistent with earlier reported literature as the absorbance at around 423 nm increased as the volume of 1 mM AgNO_3_ solution increased from 5 mL to 18 mL. However, there was an abrupt decrease in absorbance when volume of AgNO_3_was further increased to 20 mL. Thus, the optimum concentration ratio was 1:18 at which we obtained the most NPs as seen by the most intense peak at the mentioned *λ*_max_.

pH is also one of the factors that controlled the stability, shape and size of NPs [58]. The change in pH results in changes in the shape, size and stability of NPs. It has been reported that particle size decreases in higher pH which accounts for higher stability of the AgNPs and prevents further aggregation. The current study reveal that AgNPs are highly stable in basic conditions as can be seen from the UV–Vis spectra in Fig. 3 (c).

Fig. 3 (d) shows that AgNPs were quite stable from room temperature to 60 °C, but their stability dropped when heated up to 80 °C. The decrease in the absorption sharpness along with broadening of the peak with increase in temperature shows aggregation as also reported by Liu et al. [59]. The present results reveal that AgNPs are quite stable up to 60 °C but as the temperature increases up to 80 °C the size of NPs increases that results in aggregation and thus caused its stability drop.

### 3.3 Antibacterial activities

The ineffectiveness of many antibiotics are frequently attributed to the unfavorable pharmacokinetic properties of therapeutic drugs, which include limited bioavailability, poor capacity or inability to pass biological barriers, short half-life, and low chemical-physical stability [60]. Treatment of infections caused by intracellular microbes and drug-resistant strains is made more complicated with the use of antibiotics due to poor membrane transport of antibiotic, efflux pump, target modifications, and inactivation of enzyme. Therefore, average sized drugs have little effect on intracellular microbes [61]. To overcome this limitation, modified therapies using drug-containing NPs as mediators have been proposed. NPs are small particles that can be designed to target specific cells or tissues, and they can often penetrate cell membranes more easily than larger drug molecules. By encapsulating antibiotics or other drugs in NPs, it may be possible to improve their delivery to intracellular microbes or drug resistant bacteria, potentially increasing their effectiveness. As nanomaterials do not have a defined mode of action like antibiotics, they can be very helpful for combating resistance in bacterial pathogens [62]. AgNPs specifically have received considerable attention due to their ability to work as antibacterial agents [63]. The genetic toxicity which can lead to cancer depends upon NPs shape, size, surface area, surface coating, agglomeration, crystal structure and dissolution which impact the biological interactions. Small size and large surface area generate ROS which can generate H_2_O_2_ which can interact with DNA or RNA [64].

**Fig. 4:**
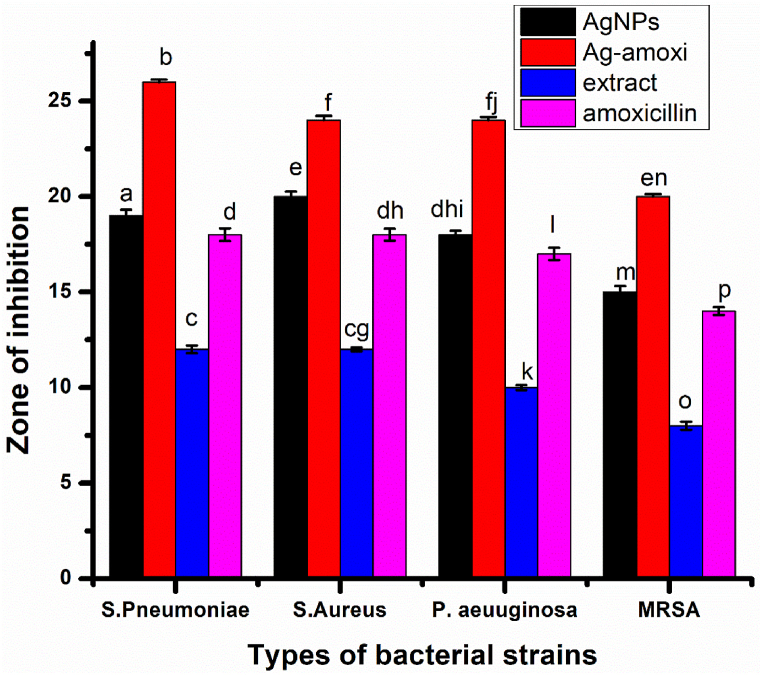
Bar graph showing the zone of inhibition of different bacterial strain by AgNPs, Ag-amoxi, plant extract and *amoxicillin*. Various letters indicating significant difference between zone of inhibition according to Duncan’s test (p < 0.05).

We have examined the antibacterial potential of AgNPs and Ag-amoxi conjugates against gram positive *Staphylococcus aureus (S.A), Streptococcus Pneuomoniae, (P.A), Methicillin Resistance Staphylococcus aureus (MRSA*), and *gram*-negative bacteria *Pseudomonas aeruginosa (P.A)* and the results obtained are shown in Fig. 4. The results reveal that Ag-amoxi conjugates showed better efficacy against all the selected bacterial strain compared to AgNPs, flavonoids extract and *amoxicillin***.**

The Ag-amoxi and AgNPs contribute to the excellent antibacterial activity due to their small size and large surface area which allow better penetration and interaction with bacteria cells compared to amoxicillin and plant extracts. The bacterial cell wall is predominantly composed of hydrophobic phospholipids, which can make it difficult for hydrophilic drugs like *amoxicillin* to penetrate the membrane and reach their targets within the bacteria. This limits the effectiveness of antibiotics against intracellular bacteria or drug resistant strains. The higher activity of the nanoparticles is due to the ability of the AgNPs to cross the hydrophobic bacteria cell walls, with the AgNPs then reacting directly or facilitating the transport of amoxicillin [53]. The strong antibacterial activity of Ag-amoxi conjugates against MRSA is particularly promising, as this strain has become increasingly resistant to multiple antibiotics and is a major public health concern. These findings suggest that Ag-amoxi conjugates could be a promising candidate for the development of new antibiotics to combat multidrug resistant bacterial infections.

### 3.4 Antioxidant properties

The DPPH assay is one of the most reliable and reproducible methods to assess the antioxidant efficacy of samples [65]. In the present study the percent inhibition of the flavonoid extract, AgNPs and Ag-amoxi conjugates towards *DPPH* were determined using different concentrations from 31.25 μg mL^−1^ to 1000 μg mL^−1^. It was found that when the concentration increased from 31.25 to 1000 μg/mL an increase in the inhibition was observed by flavonoid extract (20–74.24 %), AgNPs (20.67–70.85 %) Ag-amoxi, (24.33–79 %), and *amoxicillin* (18.69–77.68 %) compared to standard ascorbic acid (24.67–75.67 %) respectively. These results show that the Ag-amoxi has a higher antioxidant efficiency than the AgNPs, *flavonoids* extracts. The antioxidant ability of these compounds was also evaluated by measuring their IC_50_ values. The IC_50_ is the concentration of an antioxidant-containing substance required to scavenge 50 % of the initial DPPH radicals. The lower the IC_50_ value, the more potent is the substance at scavenging DPPH and this implies a higher antioxidant activity [66]. All the other values are also given in Table 1). The enhance antioxidant activity of Ag-amoxi is may be due the phytochemicals attached with surface of Ag-amoxi [11].The better IC_50_ value of Ag-amoxi compared to AgNPs may aid to the fact that this amoxicillin loaded AgNPs can be used as free radical scavenger and may scavenging the free radical generation.

**Table 1:** % Scavenging activity with IC_50_ values of AgNPs, Ag-amoxi Flavonoids extract and amoxicillin compared with standard Ascorbic acid.

S. No	Concentration (μg/mL)	% Scavenging activity				
		AgNPs	Ag-amoxi	Extract	Amoxicillin	Ascorbic acid
1	31.25	20.67*** ± 4.055	24.33** ± 4.842	20.33** ± 3.528	18.69*** ± 3.283	24.67* ± 4.333
2	62.5	39* ± 3.512	43* ± 2	39.67^ns^ ± 6.227	39*** ± 2.309	45.73* ± 6.227
3	125	48.72^ns^ ± 6.692	55.67* ± 5.207	51.0 * ± 5.51	50.69* ± 3.41	53** ± 4.041
4	250	55.61* ± 7.055	62.33* ± 8.192	60.64** ± 6.36	60.0^ns^ ± 5.774	61** ± 5.859
5	500	62.67* ± 6.227	68.53** ± 4.41	66.72** ± 4.72	70* ± 5.0	68*** ± 4.619
6	1000	70.85** ± 2.333	79*** ± 3.786	74.41*** ± 3.28	77.68* ± 4.76	75.67*** ± 3.93
	IC_50_ vales					
		46.34	45.38	42.53	58.17	34.11

Different sferics (*) show the significant difference among the compounds according to t-distribution test. * significant, ** more significant, *** most significant and (ns) not significant.

### 3.5 Anti-inflammatory activity (carrageenan hind paw model)

Inflammation is the body’s response to harmful stimuli. Heat, pain, redness, and swelling are all symptoms of an inflammatory response. Many mechanisms and mediators are involved in the inflammatory process. Inflammation usually occurs in two stages. In the first stage (1 h) when inflammation starts histamine, serotonin and bradykinin are released [67]. These mediators increase vascular permeability, allowing immune cells and fluid to move into the affected tissue to fight the source of inflammation. When carrageenan is injected subcutaneously in the mice hind paw it causes swelling (edema) and the size of the paw is increased due to severe inflammation, followed by treatment with diclofenac sodium and test compounds. Following the carrageenan injection, there may be a 5-h period during which there is an excess production of prostaglandin mediators, which are involved in the control of several physiological processes, including inflammation [68].

**Fig. 5:**
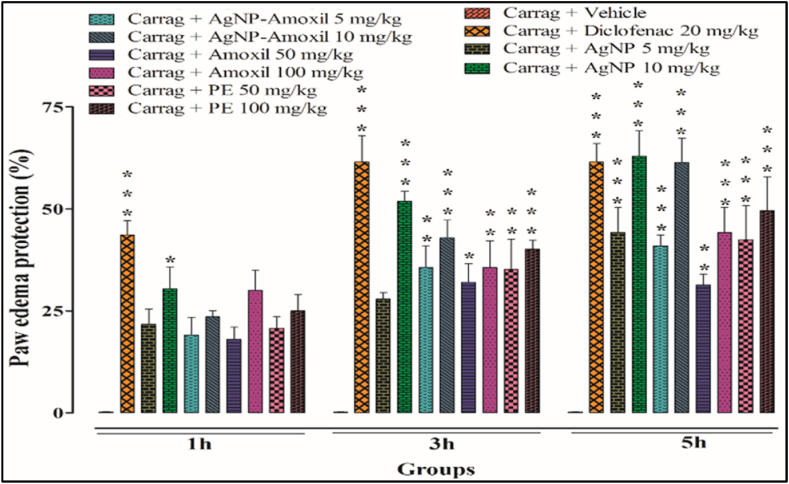
Effect of AgNPs, AgNPs-amoxi conjugate, *flavonoid’s* extract and *amoxicillin* on carrageenan induced paw edema in rats. Bars represent mean ± SEM. *p < 0.05, **p < 0.01, ***p < 0.001 compared to the vehicle treated animals. Data Analyzed by one-way ANOVA followed by Tuckey's test.

Our results reveal that diclofenac sodium as a reference standard at a dose of 20 mg kg^−1^ showed more protection during 1–3 h. However, after 5 h, the diclofenac sodium treated animal showed 56 % paw protection, while at the same time AgNPs and Ag-amoxi showed more protection i.e. 64 % and 65 %, respectively at much lower doses of 10 mg kg^−1^ compared to amoxicillin and flavonoids extract at 100 mg kg^−1^. So AgNPs and Ag-amoxi had notable benefits at both stages of inflammation and had the ability to reduce inflammation by releasing a number of mediators that may be responsible for the decrease in inflammation at both stages. All the results are shown in Fig. 5.

### 3.6 Antinociceptive activities (acetic acid writhing test)

**Fig. 6:**
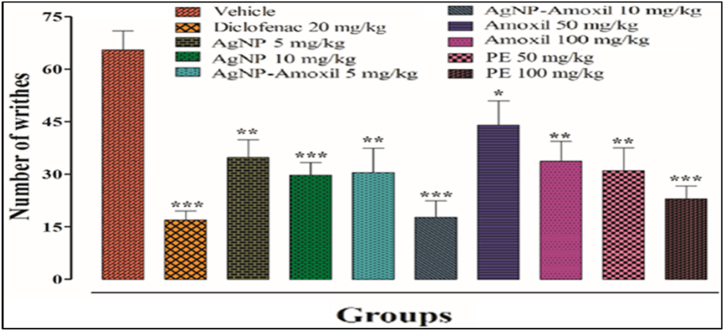
Chemically induced nociceptive pain (writhing test) Bars represent mean ± SEM. *p < 0.05, **p < 0.01***p < 0.001 compared to the vehicle treated animals Data was analyzed by one way ANOVA followed by Tuckey's test.

It has been reported that in writhing tests the signals transmitted to the central nervous system are due to pain which in turn are responsible for the release of mediators that helps to reduce the abdominal constrictions [69]. During our studies a dose dependent approach was adopted and the number of writhes of test mice injected with our compounds were counted after 20 min. Fig. 6 shows the number of writhes for mice injected with Ag-amoxi compared to AgNPs at a dose of 5 or10 mg kg^−1^, and amoxicillin and crude flavonoids extracts doses of 50 or 100 mg kg^−1^. The results show that AgNPs and the amoxi conjugate along with amoxicillin at the tested doses possessed significant (*p < 0.05, **p < 0.01, ***p < 0.001) antinociceptive activity compared to the vehicle treated animals. The technique utilised in this study is beneficial for assessing the activity of analgesics with central action, which are intended to increase the pain threshold by stimulating opioid receptors. Both peripheral and spinal pain are linked to the activation of these receptors [70].

#### 3.6.1 Hotplate test

**Fig. 7:**
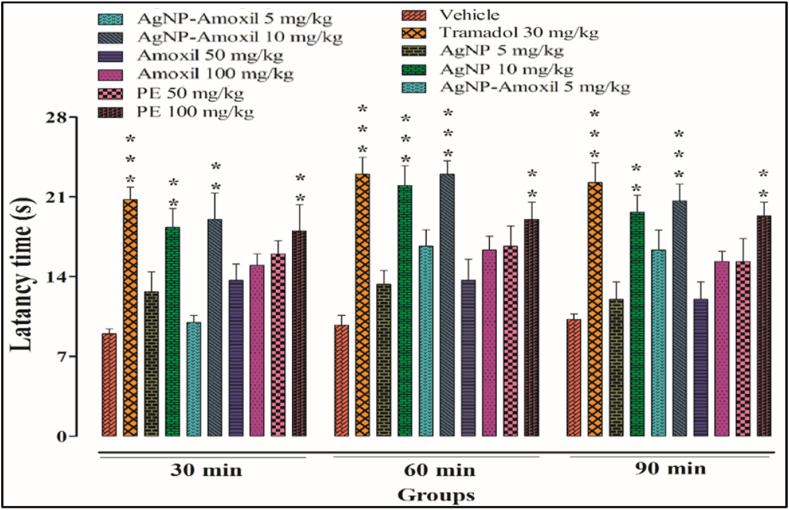
Thermally induced nociceptive pain in mice using hotplate bars represent mean ± SEM. *p < 0.05, **p < 0.01, ***p < 0.001 compared to the vehicle treated animals Data were analyzed by one way ANOVA followed by Tuckey's test.

Thermal analgesia test has been used to monitor the nociceptive pain. In the present research it has been shown that the AgNPs and the amoxi conjugates showed significant activity (p < 0.001) towards mitigating nociceptive pain at a dose of 10 mg kg^−1^ while the flavonoid extract and the amoxicillin only showed significant effects at much higher doses of 100 mg/kg. At 90 min of study all the test compounds showed maximum activities, as evident from Fig. 7.

## 4 Conclusion

We have synthesized AgNPs using crude flavonoid extract that act both as reducing and stabilizing agents and avoid the use of hazardous and toxic solvents. The nanoparticles were mostly in size ranges from 39 to 120 nm. In the next step these AgNPs were conjugated with amoxicillin, a β-Lactam drug, to get Ag-amoxi. We then evaluated antibacterial, anti-inflammatory, and antinociceptive properties of AgNPs, Ag-amoxi, flavonoids extract, and amoxicillin. The biological activity results reveal that these AgNPs and Ag-amoxi conjugates show excellent antibacterial activities against both common and resistant strain of MRSA compare to *amoxicillin* and flavonoid’s extract. Moreover, both these AgNPs and Ag-amoxi displayed better anti-inflammatory and analgesic properties than all other test compounds. The predominant in-vitro findings suggest that Ag-amoxi conjugates could be a promising candidate for the development of new antibiotics to combat multidrug resistant bacterial infections. This work could provide a better solution to the much-awaited bacterial resistance problem. However, further study is required to investigate the exact mechanism of how these nanoparticles and their drug conjugates enhance the potency of a first generation β-lactam drug.

## Data availability statement

The data generated during the current study are available from the corresponding author on request.

## CRediT authorship contribution statement

**Kamran Jalil:** Writing – original draft, Methodology, Investigation, Formal analysis. **Shabir Ahmad:** Writing – review & editing, Supervision, Conceptualization. **Nazar ul Islam:** Writing – review & editing. **Sayyar Muhammad:** Visualization, Validation, Formal analysis, Data curation. **Qudsia Jalil:** Writing – review & editing. **Asad Ali:** Writing – review & editing, Supervision.

## Declaration of competing interest

The authors declare that they have no known competing financial interests or personal relationships that could have appeared to influence the work reported in this paper.
